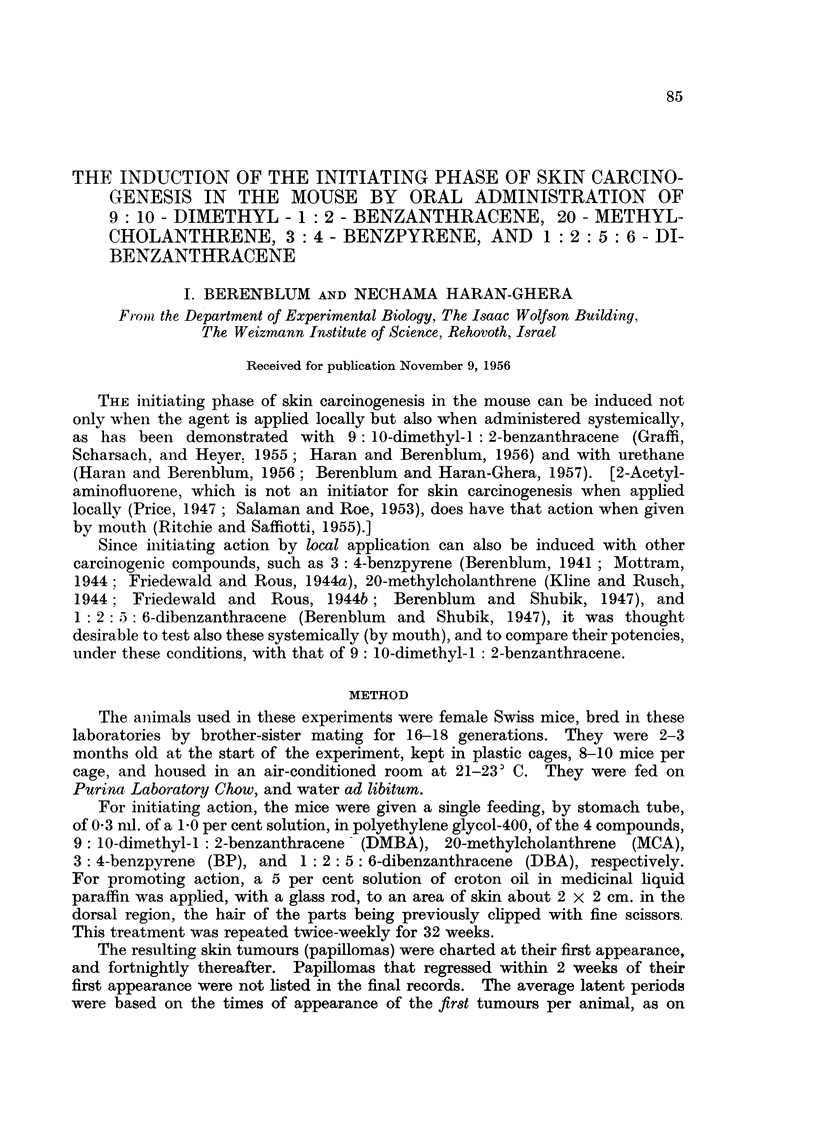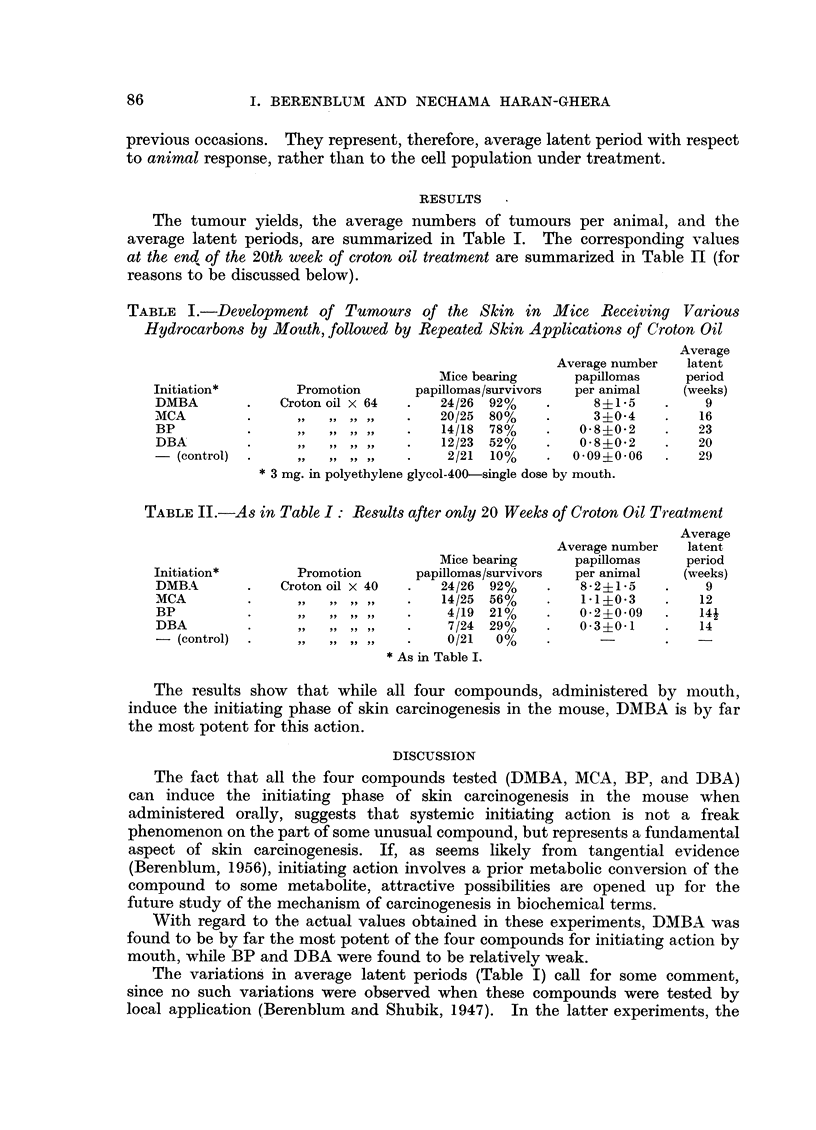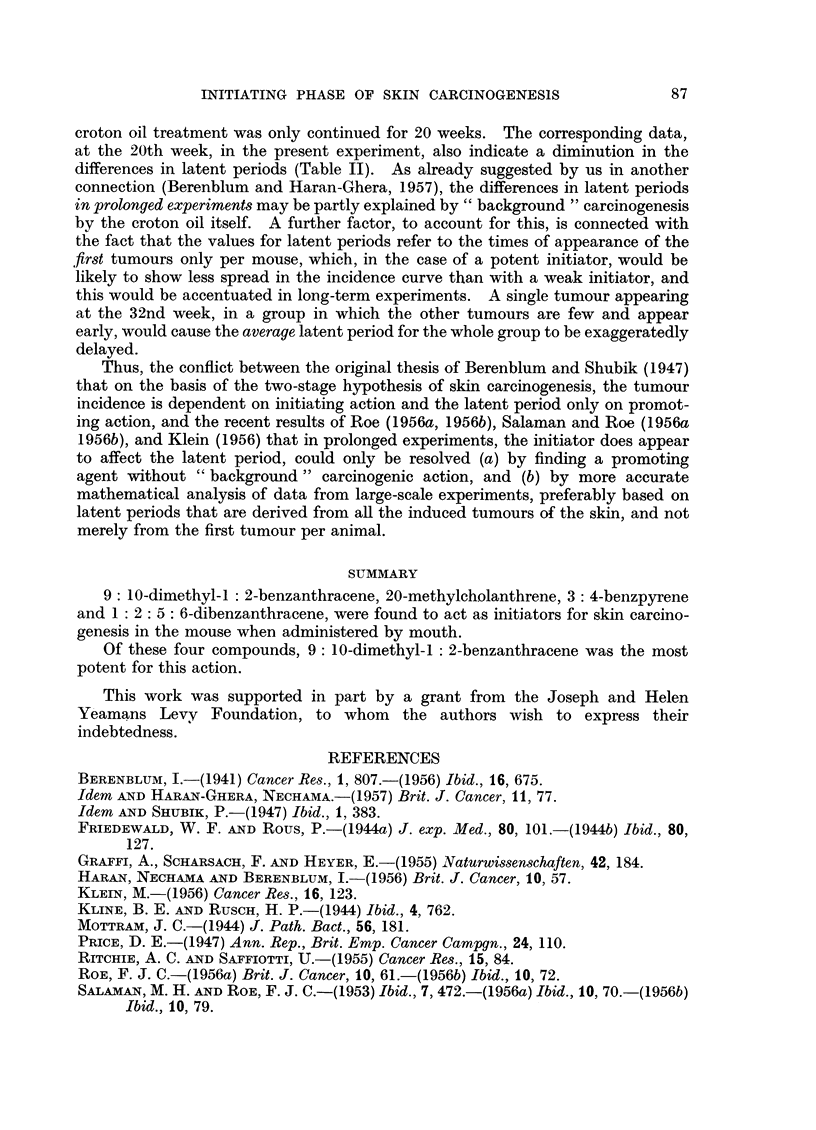# The Induction of the Initiating Phase of Skin Carcinogenesis in the Mouse by Oral Administration of 9:10 - Dimethyl - 1:2 - Benzanthracene, 20 - Methylcholanthrene, 3:4 - Benzpyrene, and 1:2:5:6 - Dibenzanthracene

**DOI:** 10.1038/bjc.1957.12

**Published:** 1957-03

**Authors:** I. Berenblum, Nechama Haran-Ghera


### THE INDUCTION OF THE INITIATING PHASE OF SKIN CARCINOGENESIS IN THE MOUSE BY ORAL ADMINISTRATION OF 9 : 10 - DIMETHYL - 1 : 2 - BENZANTHRACENE, 20 - METHYLCHOLANTHRENE, 3 : 4 - BENZPYRENE, AND 1 : 2 : 5 : 6 - DIBENZANTHRACENE

I. BERENBLUM AND NECHAMA HARAN-GHERA

*From the Department of Experimental Biology, The Isaac Wolfson Building, The Weizmann Institute of Science, Rehovoth, Israel*

Received for publication November 9, 1956

THE initiating phase of skin carcinogenesis in the mouse can be induced not only when the agent is applied locally but also when administered systemically, as has been demonstrated with 9 : 10-dimethyl-1 : 2-benzanthracene (Graffi, Scharsach, and Heyer, 1955 ; Haran and Berenblum, 1956) and with urethane (Haran and Berenblum, 1956 ; Berenblum and Haran-Ghera, 1957). [2-Acetylaminofluorene, which is not an initiator for skin carcinogenesis when applied locally (Price, 1947 ; Salaman and Roe, 1953), does have that action when given by mouth (Ritchie and Saffiotti, 1955).]

Since initiating action by *local* application can also be induced with other carcinogenic compounds, such as 3 : 4-benzpyrene (Berenblum, 1941 ; Mottram, 1944 ; Friedewald and Rous, 1944*a*), 20-methylcholanthrene (Kline and Rusch, 1944 ; Friedewald and Rous, 1944*b* ; Berenblum and Shubik, 1947), and 1 : 2 : 5 : 6-dibenzanthracene (Berenblum and Shubik, 1947), it was thought desirable to test also these systemically (by mouth), and to compare their potencies, under these conditions, with that of 9 : 10-dimethyl-1 : 2-benzanthracene.

### METHOD

The animals used in these experiments were female Swiss mice, bred in these laboratories by brother-sister mating for 16–18 generations. They were 2–3 months old at the start of the experiment, kept in plastic cages, 8–10 mice per cage, and housed in an air-conditioned room at 21–23° C. They were fed on *Purina Laboratory Chow*, and water *ad libitum.*

For initiating action, the mice were given a single feeding, by stomach tube, of 0·3 ml. of a 1·0 per cent solution, in polyethylene glycol-400, of the 4 compounds, 9 : 10-dimethyl-1 : 2-benzanthracene (DMBA), 20-methylcholanthrene (MCA), 3 : 4-benzpyrene (BP), and 1 : 2 : 5 : 6-dibenzanthracene (DBA), respectively. For promoting action, a 5 per cent solution of croton oil in medicinal liquid paraffin was applied, with a glass rod, to an area of skin about 2 × 2 cm. in the dorsal region, the hair of the parts being previously clipped with fine scissors. This treatment was repeated twice-weekly for 32 weeks.

The resulting skin tumours (papillomas) were charted at their first appearance, and fortnightly thereafter. Papillomas that regressed within 2 weeks of their first appearance were not listed in the final records. The average latent periods were based on the times of appearance of the *first* tumours per animal, as on previous occasions. They represent, therefore, average latent period with respect to *animal* response, rather than to the cell population under treatment.

### RESULTS

The tumour yields, the average numbers of tumours per animal, and the average latent periods, are summarized in Table I. The corresponding values *at the end of the 20th week of croton oil treatment* are summarized in Table II (for reasons to be discussed below).

TABLE I.—*Development of Tumours of the Skin in Mice Receiving Various Hydrocarbons by Mouth, followed by Repeated Skin Applications of Croton Oil*

| Initiation* | Promotion | Mice bearing papillomas/survivors | | Average number papillomas per animal | Average latent period (weeks) |
|---|---|---|---|---|---|
| DMBA | Croton oil × 64 | 24/26 | 92% | 8±1·5 | 9 |
| MCA | ,, ,, ,, ,, | 20/25 | 80% | 3±0·4 | 16 |
| BP | ,, ,, ,, ,, | 14/18 | 78% | 0·8±0·2 | 23 |
| DBA | ,, ,, ,, ,, | 12/23 | 52% | 0·8±0·2 | 20 |
| — (control) | ,, ,, ,, ,, | 2/21 | 10% | 0·09±0·06 | 29 |

* 3 mg. in polyethylene glycol-400—single dose by mouth.

TABLE II.—*As in Table I : Results after only* 20 *Weeks of Croton Oil Treatment*

| Initiation* | Promotion | Mice bearing papillomas/survivors | | Average number papillomas per animal | Average latent period (weeks) |
|---|---|---|---|---|---|
| DMBA | Croton oil × 40 | 24/26 | 92% | 8·2±1·5 | 9 |
| MCA | ,, ,, ,, ,, | 14/25 | 56% | 1·1±0·3 | 12 |
| BP | ,, ,, ,, ,, | 4/19 | 21% | 0·2±0·09 | 14½ |
| DBA | ,, ,, ,, ,, | 7/24 | 29% | 0·3±0·1 | 14 |
| — (control) | ,, ,, ,, ,, | 0/21 | 0% | — | — |

* As in Table I.

The results show that while all four compounds, administered by mouth, induce the initiating phase of skin carcinogenesis in the mouse, DMBA is by far the most potent for this action.

### DISCUSSION

The fact that all the four compounds tested (DMBA, MCA, BP, and DBA) can induce the initiating phase of skin carcinogenesis in the mouse when administered orally, suggests that systemic initiating action is not a freak phenomenon on the part of some unusual compound, but represents a fundamental aspect of skin carcinogenesis. If, as seems likely from tangential evidence (Berenblum, 1956), initiating action involves a prior metabolic conversion of the compound to some metabolite, attractive possibilities are opened up for the future study of the mechanism of carcinogenesis in biochemical terms.

With regard to the actual values obtained in these experiments, DMBA was found to be by far the most potent of the four compounds for initiating action by mouth, while BP and DBA were found to be relatively weak.

The variations in average latent periods (Table I) call for some comment, since no such variations were observed when these compounds were tested by local application (Berenblum and Shubik, 1947). In the latter experiments, the croton oil treatment was only continued for 20 weeks. The corresponding data, at the 20th week, in the present experiment, also indicate a diminution in the differences in latent periods (Table II). As already suggested by us in another connection (Berenblum and Haran-Ghera, 1957), the differences in latent periods *in prolonged experiments* may be partly explained by " background " carcinogenesis by the croton oil itself. A further factor, to account for this, is connected with the fact that the values for latent periods refer to the times of appearance of the *first* tumours only per mouse, which, in the case of a potent initiator, would be likely to show less spread in the incidence curve than with a weak initiator, and this would be accentuated in long-term experiments. A single tumour appearing at the 32nd week, in a group in which the other tumours are few and appear early, would cause the *average* latent period for the whole group to be exaggeratedly delayed.

Thus, the conflict between the original thesis of Berenblum and Shubik (1947) that on the basis of the two-stage hypothesis of skin carcinogenesis, the tumour incidence is dependent on initiating action and the latent period only on promoting action, and the recent results of Roe (1956*a*, 1956*b*), Salaman and Roe (1956*a* 1956*b*), and Klein (1956) that in prolonged experiments, the initiator does appear to affect the latent period, could only be resolved (*a*) by finding a promoting agent without " background " carcinogenic action, and (*b*) by more accurate mathematical analysis of data from large-scale experiments, preferably based on latent periods that are derived from all the induced tumours of the skin, and not merely from the first tumour per animal.

### SUMMARY

9 : 10-dimethyl-1 : 2-benzanthracene, 20-methylcholanthrene, 3 : 4-benzpyrene and 1 : 2 : 5 : 6-dibenzanthracene, were found to act as initiators for skin carcinogenesis in the mouse when administered by mouth.

Of these four compounds, 9 : 10-dimethyl-1 : 2-benzanthracene was the most potent for this action.

This work was supported in part by a grant from the Joseph and Helen Yeamans Levy Foundation, to whom the authors wish to express their indebtedness.

### REFERENCES

BERENBLUM, I.—(1941) *Cancer Res.*, **1**, 807.—(1956) *Ibid.*, **16**, 675.

*Idem* AND HARAN-GHERA, NECHAMA.—(1957) *Brit. J. Cancer*, **11**, 77.

*Idem* AND SHUBIK, P.—(1947) *Ibid.*, **1**, 383.

FRIEDEWALD, W. F. AND ROUS, P.—(1944*a*) *J. exp. Med.*, **80**, 101.—(1944*b*) *Ibid.*, **80**, 127.

GRAFFI, A., SCHARSACH, F. AND HEYER, E.—(1955) *Naturwissenschaften*, **42**, 184.

HARAN, NECHAMA AND BERENBLUM, I.—(1956) *Brit. J. Cancer*, **10**, 57.

KLEIN, M.—(1956) *Cancer Res.*, **16**, 123.

KLINE, B. E. AND RUSCH, H. P.—(1944) *Ibid.*, **4**, 762.

MOTTRAM, J. C.—(1944) *J. Path. Bact.*, **56**, 181.

PRICE, D. E.—(1947) *Ann. Rep., Brit. Emp. Cancer Campgn.*, **24**, 110.

RITCHIE, A. C. AND SAFFIOTTI, U.—(1955) *Cancer Res.*, **15**, 84.

ROE, F. J. C.—(1956*a*) *Brit. J. Cancer*, **10**, 61.—(1956*b*) *Ibid.*, **10**, 72.

SALAMAN, M. H. AND ROE, F. J. C.—(1953) *Ibid.*, **7**, 472.—(1956*a*) *Ibid.*, **10**, 70.—(1956*b*) *Ibid.*, **10**, 79.